# Phylogenetic analysis and molecular characteristics of seven variant Chinese field isolates of PRRSV

**DOI:** 10.1186/1471-2180-10-146

**Published:** 2010-05-20

**Authors:** Chengmin Wang, Bin Wu, Said Amer, Jing Luo, Hongmei Zhang, Yunhai Guo, Guoying Dong, Baohua Zhao, Hongxuan He

**Affiliations:** 1 National Research Center for Wildlife Born Diseases, Institute of Zoology, Chinese Academy of Sciences, Beijing 100101, China; 2 College of Life Science, Hebei Normal University, Shijiazhuang, Hebei, 050060 China; 3 Department of Life Science, Heze College, Heze, Shandong Province 274015 China; 4 Department of Zoology, Faculty of Science, Kafr El-Sheikh 33516 Egypt

## Abstract

**Background:**

Porcine reproductive and respiratory syndrome (PRRS) has now been widely recognized as an economically important disease. The objective of this study was to compare the molecular and biological characteristics of porcine reproductive and respiratory syndrome virus (PRRSV) field isolates in China to those of the modified live virus (MLV) PRRS vaccine and its parent strain (ATCC VR2332).

**Results:**

Five genes (GP2, GP3, GP4, GP5 and NSP2) of seven isolates of PRRSV from China, designated LS-4, HM-1, HQ-5, HQ-6, GC-2, GCH-3 and ST-7/2008, were sequenced and analyzed. Phylogenetic analyses based on the nucleotide sequence of the ORF2-5 and NSP2 showed that the seven Chinese isolates belonged to the same genetic subgroup and were related to the North American PRRSV genotype. Comparative analysis with the relevant sequences of another Chinese isolate (BJ-4) and North American (VR2332 and MLV) viruses revealed that these isolates have 80.8-92.9% homology with VR-2332, and 81.3-98.8% identity with MLV and 80.7-92.9% with BJ-4. All Nsp2 nonstructural protein of these seven isolates exhibited variations (a 29 amino acids deletion) in comparison with other North American PRRSV isolates. Therefore, these isolates were novel strain with unique amino acid composition. However, they all share more than 97% identity with other highly pathogenic Chinese PRRSV strains. Additionally, there are extensive amino acid (aa) mutations in the GP5 protein and the Nsp2 protein when compared with the previous isolates.

**Conclusions:**

These results might be useful to study the genetic diversity of PRRSV in China and to track the infection sources as well as for vaccines development.

## Background

Porcine reproductive and respiratory syndrome virus (PRRSV) is recognized as one of the major infective agents in the pig industry worldwide since its appearance in the 1980s. It was first diagnosed in the USA in 1987 [1], immediately found in Europe, soon disseminated to the rest of the world [2]. The disease is characterized by reproductive failure in pregnant sows and respiratory distress particularly in suckling piglets, thereupon getting its name. PRRSV is a single-stranded positive RNA virus and a member of the family Arteriviridae in the order of Nidovirales [3]. Based on phylogenetic analyses of different virus isolates around the world, PRRSV can be differentiated into two genotypes: Type I, represented by the European prototype Lelystad strain LV, and Type II, the prototype being the Northern American ATCC strain VR2332. Chinese isolates were assigned as members of the genotype II [4]. Extensive molecular studies show that PRRSV is highly variable in antigenicity, virulence and sequence diversity [5,6].

PRRSV is a small, enveloped, single positive-stranded RNA virus including a genome of about 15 kb, encoding nine ORFs [2,7,8]. The PRRSV genome is comprised of two polymerase genes, ORF1a and 1b, and seven structural genes, ORF2a, 2b, 3, 4, 5, 6, and 7 [9]. ORF1a and ORF1b constitutes approximately 75% of the viral genome, and are characterized by a process of ribosomal frame shifting translated into a large polyprotein; which by self-cleavage gives rise to the non-structural proteins (NSPs) including the RNA-dependent RNA polymerase [10]. Open reading frames 2a, 3, 4 and 5 all encode glycosylated proteins, designated GP2a, GP3, GP4, and GP5, respectively [7,11]. The newly defined ORF2b encodes the smallest protein of the virus particle designated GP2b [8,12]. ORF7 encodes the non-glycosylated nucleocapsid protein (N), constituting 20-40% of the protein content of the virion [8,13,14]. ORF6 encodes the likewise non-glycosylated matrix protein (M) [8,12]. Heterodimers constituted by GP5 and M have been found in the endoplasmic reticulum of infected cells [14], and have been suggested to be involved in virus-host cell receptor interaction [15]. A rapid genetic divergence of PRRSV was revealed by an experiment of serial in vivo passage of a PRRSV strain [16] and by an analysis of naturally infected pigs. The presence of genetically divergent viruses in a swine population may complicate the disease control by vaccination, because the PRRSV vaccine efficacy is reduced when the challenge virus is a virus of a different genotype [17] or of a different phylogenetic cluster within the same genotype [18].

In China the first outbreak of PRRS was recorded in 1995 which encountered almost all provinces (include Hong Kong). Due to its economic impact in China, the disease has been recognized as one of the most severe viral diseases for pig farms. The first Chinese strain of PRRSV was isolated in 1996, and the complete genome sequence of the Chinese PRRSV isolate BJ-4 was first reported in 2001 [19]. Highly pathogenic PRRSV is the causative agent of porcine high fever syndrome and characterized by high fever and high death rates in pigs of all ages. Since May 2006, the highly pathogenic PRRSV has emerged in China. Recently, the genomic characteristics of two other Chinese isolates of PRRSV were described with comparisons to some American and European isolates [4]. It has been documented that PRRSV strains differ in virulence [20-23] and vary genetically [24,25]. Concerns that vaccine strains or derivatives of the vaccine strains may induce disease continue to be discussed [26-28]. The objective of this research was to compare the genetic and molecular characteristics of seven Chinese PRRSV field isolates to that of a known high-virulence PRRSV isolate (BJ-4), the Ingelvac PRRS MLV vaccine, and the parent strain of the vaccine (ATCC VR2332). The results inferred from this study might be useful for infection tracking as well as for vaccines development.

## Results and discussion

For a long time, outbreaks of highly pathogenic (acute, atypical) PRRS in many Chinese territories have been attributed to the highly virulent Chinese-type PRRSV (H-PRRSV) strains. From January to July 2007, 39455 morbid pigs died among 143,221 infected pigs according to the administrative files [29]. New types of PRRSV variants with high pathogenicity were identified in China was responsible for severe impact on pig industry as well as food safety [30]. Concurrently, this Chinese variant of PRRSV was detected in Vietnam where it caused a serious epidemic [31].

**Figure 1:**
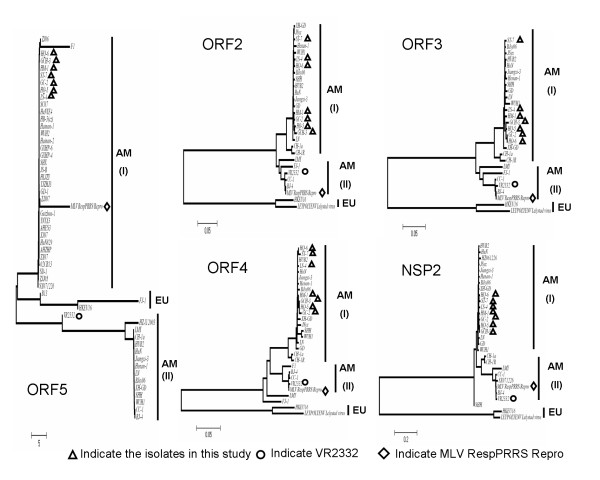
**Phylogenetic trees of the nucleotide sequences for the ORF2, ORF3, ORF4, ORF5, and NSP2 genes of the Chinese isolates (LS-4, HM-1, HQ-5, HQ-6, GC-2, GCH-3 and ST-7) and related reference viruses**. The evolutionary relationships among these viruses were estimated by the neighbor-joining method with 100 bootstraps by using PHYLIP version 3.67. Alignments of each influenza virus sequence were generated using program Clustal W. The compared sequence regions were as follows: (771 bp) of *ORF2*, (777 bp) of *ORF3*; (552bp) of *ORF4*, (603 bp) of *ORF5 *and (893 bp) of *NSP2*. Black triangles indicate the virus isolates were isolated in this study. Two main subgroups of PRRSV isolates (I and II) are indicated for ORF2-5 and NSP2 genes.

In this study, LS-4, HM-1, HQ-5, GCH-3, GC-2, HQ-6 and ST-7 strains were isolated from Hebei province. Homology search and phylogenetic analyses indicated that the sequences of seven isolates belong to the American (AM) genotype (Figure 1). Two subgroups were classified based on ORF2, ORF3, ORF4, ORF5 and NSP2 genes of Chinese American genotype isolates, and named as subgroup AM-I and AM-II (Figure 1). These seven isolates clustered to the subgroup AM-I for ORF2-5 and NSP2, whereas the Chinese isolates BJ-4, VR2332 and MLV were affiliated with subgroup AM-II based on ORF2-4 and NSP2. MLV joined the seven isolates into the subgroup (AM-I) based on ORF5 genes and show a higher evolutionary divergence (2.372-2.429) at the nucleotide acid level (Additional file 1). The results have indicated that all seven Chinese virus isolates formed a subgroup in the North American genotype, but the BJ-4 isolate was assigned to another subgroup closely related to the vaccine strain RespPRRS/Repro, suggesting that these strains may not be evolved from a revertant of the vaccine virus.

**Figure 2:**
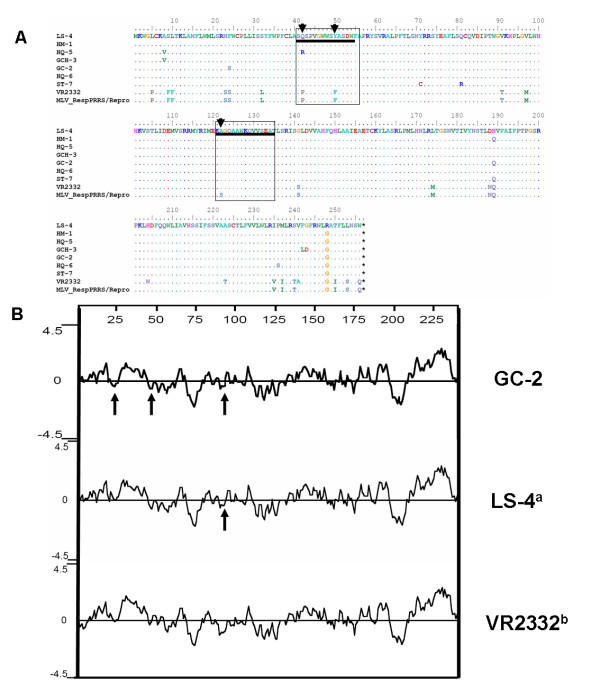
**The deduced amino acid sequence comparison and hydrophobicity profiles of the gp2 proteins between the 7 isolates and reference viruses**. A, The deduced amino acid sequence comparison of the gp2 proteins between the 7 isolates from China (GenBank accession no. EU017510, EU177103, EU177108, EU177117, EU255922, EU642603 and EU653015) and another Chinese isolates (BJ-4) (GenBank accession no. AF331831), VR2332 (GenBank accession no. EF536003) and MLV (GenBank accession no. AF159149) available in GenBank. Only the amino acids different from those in the consensus sequence are indicated. The black boxed residues indicate the immunodominant B-cell linear epitopes AA position sites. B, Hydrophobicity profiles of ORF2 generated by the Kyte and Doolittle method using DNAstar program. Major areas of difference are indicated by arrows. a, LS-4 was a representative of other five isolates because the same plots were shown for ST-7, GCH-3, HM-1, HQ-5, HQ-6 and LS-4. b, VR2332 was a representative of other three reference virus because the same plots were shown for BJ-4 and MLV.

The glycoprotein 2 (gp2) is a minor component of the PRRSV envelope [32] with 2 B-cell linear epitopes, whose reactive peptides comprise regions at amino acid positions 41-55 and 121-135 within the ORF2 sequence [33]. In the present study, those seven Chinese isolates have a lower evolutionary divergence (0.086-0.107) with VR-2332, and (0.077-0.098) with MLV and BJ-4 for ORF2 (Additional file 2). In comparison to VR2332 and MLV, two AA mutations were found at positions 42 (P→Q/R) and 50 (F→Y) (Figure 2A) and have influenced the hydrophobicity of the reactive peptides 41-55 (Figure 2B). However, another mutation at AA position 122 (S→A) did not affect the hydrophobicity of the reactive peptides 121-135 (Figure 2B). In addition, other AA mutations such as positions 23(S→N), 24 (S→F), 91 (T→K) and 97 (M→V) affect obviously the hydrophobicity of gp2 protein, which might alter the antigenic activity of gp2 (Additional file 3).

**Figure 3:**
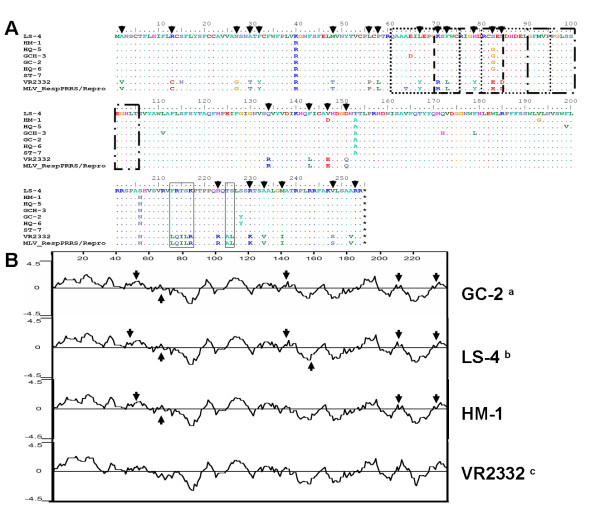
**The deduced amino acid sequence comparison and hydrophobicity profiles of the gp3 proteins between the 7 isolates and reference viruses**. A, deduced amino acid sequence comparison of the gp3 proteins between the 7 isolates from China (GenBank accession no. EU017511, EU177104, EU177109, EU177118, EU255923, EU366149 and EU439254) and another Chinese isolates (BJ-4) (GenBank accession no. AF331831), VR2332 (GenBank accession no. EF536003) and MLV (GenBank accession no. AF159149) available in GenBank. Only the amino acids different from those in the consensus sequence are indicated. The black boxed residues indicate the difference AA position sites. B, Hydrophobicity plots of ORF3 generated by the Kyte and Doolittle method using by DNAstar program. Major areas of difference are indicated by arrows. a, GC-2 was a representative of other three isolates because the same plots were shown for GCH-3, HQ-5 and HQ-6. b, LS-4 was a representative of other two isolates because the same plots were shown for LS-4 and ST-7. c, VR2332 was a representative of other two reference virus because the same plots were shown for VR2332, BJ-4 and MLV.

The highly glycosylated ORF3-encoded protein is the second most variable PRRSV protein [7], showing approximately an evolutionary divergence of 0.144-0.157 with VR-2332, MLV and BJ-4 (Additional file 4). Marcelo et al (2006) reported that 4 overlapping consecutive peptides (AA positions 61-105) were strongly immunoreactive with 85-100% of the tested sera. Those peptides were predicted to be located in the most hydrophilic region within the ORF3 sequence. Marcelo et al suggested that this region might be considered as an important immuno-dominant domain of the gp3 of North American strains of PRRSV [30]. In this study, eight AA mutations were detected at position 64 to 85 within four overlapping consecutive peptides (Figure 3A). Additionally, two novel epitopes located at 73-87aa (named GP3EP3) and 66-81aa (named GP3EP7) were identified in the gp3 of Chinese isolate (US-type) of PRRSV [34]. These authors found that the minimum amino acid sequence requirements for epitope binding were 74-85aa (W^74^CRIGHDRCGED^85^) and 67-74aa (Y^67^EPGRSLW^74^) using mutation deletion analysis. Especially these mutations at AA positions 64 (T→A), 67 (Y→L), 71 (R→K), 73 (L→F), 79 (Y→H), 83(E→S/G) and 85(D→N) affect obviously the hydrophobicity of gp3 protein comparing to VR2332 and MLV (Figure 3B). Furthermore, antigenic index analysis was predicted to observe the changes of antigenic characterization by DNAstar program (DNAStar Lasergene V7.10). The changes of the antigenic index were found to be at AA positions 60-90 (Additional file 5). Additional substitutions were observed at AA positions 1 to 10, 130 to 150 and 205-230, where AA mutations at these regions occurred correspondingly (Additional file 5). However, further investigations are needed to determine the effects of such mutations on the host-virus interaction.

**Figure 4:**
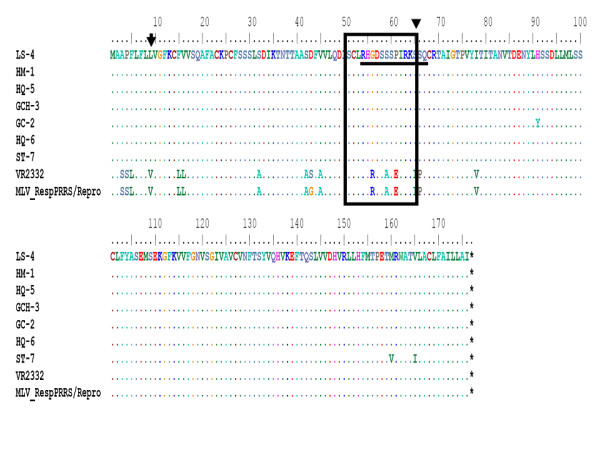
**The deduced amino acid sequence comparison and hydrophobicity profiles of the gp4 proteins between the 7 isolates and reference viruses**. Deduced amino acid sequence comparison of the gp4 proteins between the 7 isolates from China (GenBank accession no. EU017512, EU177105, EU177110, EU177119, EU177113, EU255926 and EU366150) and another Chinese isolates (BJ-4) (GenBank accession no. AF331831), VR2332 (GenBank accession no. EF536003) and MLV (GenBank accession no. AF159149) available in GenBank. Only the amino acids different from those in the consensus sequence are indicated. The black boxed residues indicate the difference AA position sites.

The glycoprotein 4 (gp4) is also a minor component of the PRRSV envelope [7] and a typical class I membrane protein [10]. Sequences of ORF4derived from the tested seven isolates showed an evolutionary divergence of 0.095-0.108 with VR2332, MLV and 0.102-0.114 with BJ-4 (Additional file 6). Previous study revealed that the gp4 protein of a North American strain of PRRSV contained one immunodominant domain, comprising amino acid residues 51-65 [33]. In our study, those mutations at AA positions 9(V→L), 32(A→S), 56 (R→G), 59 (A→S), 61 (E→P) and 78(V→I) obviously affect the hydrophobicity of gp4 protein compared to VR2332 and MLV (Figure 4). The core of a neutralization domain of the glycoprotein encoded by ORF4 of Lelystad virus and recognized by MAbs consists of amino acids 59 to 67 and is located at the most variable region of the protein [35]. The two mutations of positions 59 (A→S) and 61 (E→P) exactly located within this region and may affect the antigenicity of Chinese isolates in the present study. Antigenic index analysis revealed that seven antigenic changes for virus isolate LS-4, GCH-3, HM-1, HQ-5, HQ-6 and ST-7 and five antigenic changes for virus isolate GC-2 were observed (Additional file 7). However, further studies are necessary to demonstrate whether the putative linear epitope identified in the present study is recognized by neutralizing antibodies.

Glycoprotein 5 (gp5) is one of the major structural proteins encoded by PRRSV and forms disulfide-linked heterodimers with M protein in the viral envelope [7]. The ORF5 of PRRSV encodes a 24.5-26 kDa envelope protein with a characteristic hydropathy profile and putative glycosylation sites [11,14,36]. Amplicons of ORF5 genes derived from the 7 tested isolates had the same size of 603 bp (deduced amino acids are 201). The sequence alignments indicated that they had an identity of 99-100% at the nucleotide level and 98-100% at the amino acid level between MLV and BJ-4. However, the deduced amino acid sequence comparison indicated that those isolates show an higher evolutionary divergence of 2.372-2.429 with VR-2332 and MLV,3.314-3.471 with BJ-4 (Additional file 1), and displayed considerable genetic variation.

**Figure 5:**
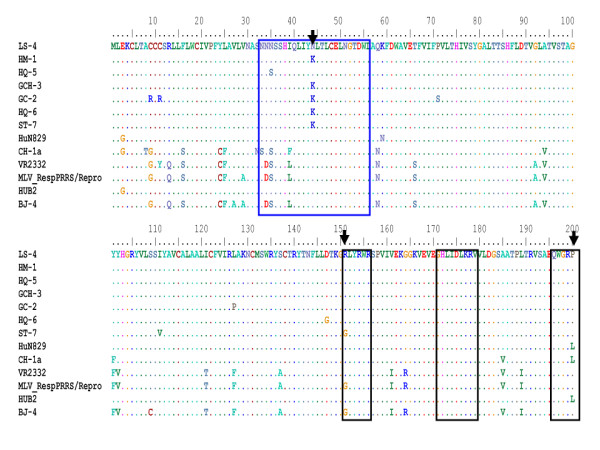
**The deduced amino acid sequence comparison of the gp5 proteins between the 7 isolates and reference viruses**. The deduced amino acid sequence comparison of the gp5 proteins between the 7 isolates from China (GenBank accession no. EU075303, EU177106, EU439252, EU177120, EU177114, EU255925 and EU366151) and Chinese isolates (BJ-4) (GenBank accession no. AF331831), HUB829(GenBank accession no. EU399853), CH-1a (GenBank accession no. AY032626), HUB2 (GenBank accession no. EF112446), VR2332 (GenBank accession no. EF536003) and MLV (GenBank accession no. AF159149) available in GenBank. Only the amino acids different from those in the consensus sequence are indicated. The black boxed residues indicate the Linear B epitope sites.

Porcine reproductive and respiratory syndrome virus (PRRSV) glycoprotein 5 (GP5) is the most abundant envelope glycoprotein and a major inducer of neutralizing antibodies in vivo, containing three putative N-linked glycosylation sites (N34, N44, and N51), where a major neutralization epitope [37] is located. Plagemann et al. [38] also used peptide mapping to show that the major neutralization epitope of PRRSV is located to the middle of the GP5 ectodomain (aa 36-52). This neutralization epitope is flanked by multiple N-linked glycosylation sites, which are probably important for correct folding, targeting, and biological activity of the protein. The loss of these N-linked glycosylation sites enhances both the sensitivity of these viruses to in vitro neutralization and the immunogenicity of the nearby neutralization epitope. In this study, only gp5 proteins of isolate LS-4 and HQ-5 had these three N-linked glycosylation sites, while other five isolates (GCH-3, HM-1, HQ-6, GC-2 and ST-7) had two N-linked glycosylation sites (N34 and N51) because of mutation of N44 glycosylation site (N→K). It has been demonstrated that the retention of N44 was very crucial for infection of PRRSV [37,39]. However, the biological characterization of those N44 deletion isolates should be further analyzed in future work. These results have indicated the sensitivity of most Chinese virus isolates to neutralization by PRRSV-specific antibodies after vaccination. In another study, a neutralizing epitope in the ectodomain of gp5 has been previously described [40]. The core sequence of this neutralizing epitope (H38, Q40, I42, Y43 and N44) was present in gp5 proteins of isolates LS-4 and HQ-5, while other isolates had only shown a mutant epitope (H38, Q40, I42, Y43 and K44) (Figure 5). It is suggested that mutation variants of N44 glycosylation site loss have great significance for development of PRRSV vaccines of enhanced protective efficacy. Three minimal epitopes (RLYRWR, EGHLIDLKRV and QWGRL) were precisely defined in the C terminus of GP5 protein and were highly conserved among the North American type isolates [41]. The sequence "QWGRL" might be a characteristic of highly pathogenic PRRSV, while corresponding AA position of low pathogenic PRRSV show "RWGRL" [41]. A mutation (R151G) of ST-7 isolate was identical to MLV and BJ-4, while other six isolates were the same with VR2332, HUB2, CH-1a and HuN829 (Figure 5).

**Figure 6:**
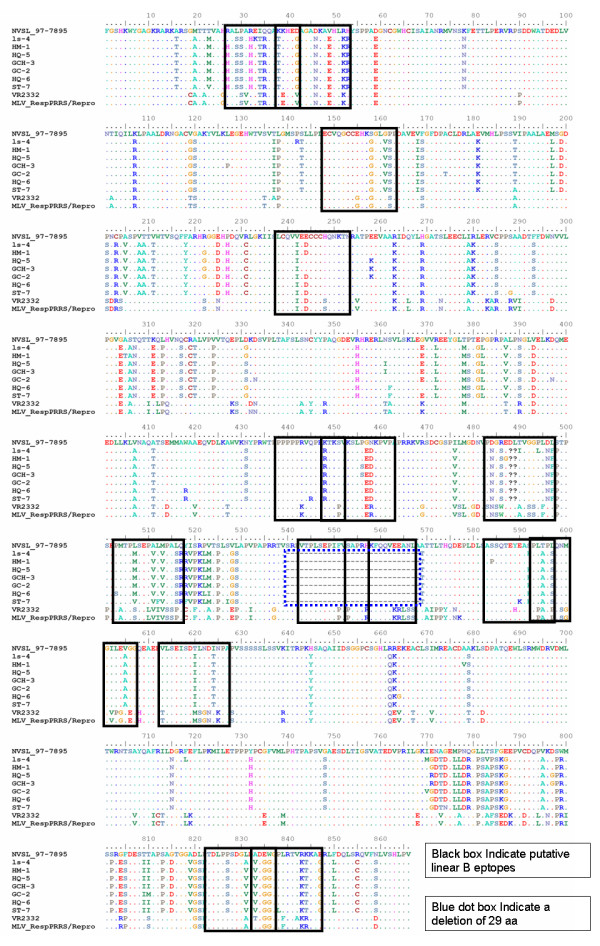
**Amino acid sequence comparison of the nsp2 proteins between the 7 isolates from China (GenBank accession no. **EU075304, EU177102, EU255920, EU669820, EU255919, EU653014** and **EU642604) **and another isolates NVSL 97-7895 (GenBank accession no. **AY545985), **VR2332 (GenBank accession no. **EF536003) **and MLV (GenBank accession no. **AF159149) **available in GenBank**. Dots indicate amino acids identical to LS-4 and deletions are indicated by dashes (--). The black boxed residues indicate the putative linear B epitopes. The blue dot boxed indicate a deletion of AA.

Phylogenetic analysis based on the deduced amino acid sequences of Nsp2 gene obtained during this study and those of isolates VR2332, and MLV strains retrieved from GenBank, indicated that all the seven Nsp2 sequences belonged to the North American genotype. Comparison between seven Chinese isolates and both VR-2332 MLV and BJ-4 showed 0.275-0.281, 0.272-0.278 and 0.275-0.283 nucleotide identity (Additional file 8), respectively. Remarkably, compared to the VR-2332 and MLV strain, analysis of the partial Nsp2 sequences revealed that a 30-aa deletion of a fragment containing a major hydrophilic region had occurred from residues 540 to 569 (Figure 6), which was also previously reported [42,43]. Some evidences have pointed to the conclusion that the highly pathogenic PRRSV with the 30-aa deletion in Nsp2 is the causative agent of atypical PRRS in China [42,44,45]. On the contrary, another research has reported that the 30-amino-acid deletion in the Nsp2 of highly pathogenic porcine reproductive and respiratory syndrome virus emerging in China is not related to its virulence [46].

The Nsp2 protein has been shown to be highly variable among arteriviruses, with similarities observed only in the amino- and carboxy-terminal domains whereas the central region of the protein varies in both length and amino acid composition [47]. Interestingly, the Nsp2 protein was found to contain the highest frequency of immunogenic epitopes including positions 27-42, 37-52, 483-497, 503-517,823-837 and 833-847, when compared to reference virus strains examined in this study (Figure 6). In addition, these immuno-dominant B-cell epitopes were scattered along the protein sequence, and most of them were localized within predicted hydrophilic regions of the protein by predicting hydropathy Kyte-Doolittle method (Additional file 9). These results were not unexpected since hydrophilic amino acid sequences are likely to be exposed on the surface of the protein and thus may be more easily recognized by B-lymphocytes. A previous report has also demonstrated the occurrence of a cluster of B-cell epitopes in Nsp2 of an EUtype PRRSV isolate and a north America PRRSV isolate, NVSL 97-7895 strain [33,48].

## Conclusions

In conclusion, this study presented detailed molecular and phylogenetic analyses for seven field isolates of PRRSV from China. The collected results revealed that the highly pathogenic PRRSV variants with the 30-aa deletion in Nsp2 were still the dominating viruses in China. The genetic diversity of PRRSV strain existed in the field in China. These results might be useful for the origin and genetic diversity of PRRSV Chinese isolates and the development of vaccine candidates in the future.

## Methods

### Cell culture and viruses

Swine Alveolar Macrophages (SAM) were obtained from about 4 week-old pigs as previously described [49]. The cells were cultured in RPMI-1640 medium supplemented with 10% fetal bovine serum and antibiotics (25 U/ml penicillin, 25 μg/ml streptomycin, 40 μg/ml gentamicin, 25 μg/ml neomycin and 300 U/ml polymyxin). Monkey kidney cell line, MARC-145 [50], was cultured in Eagle's minimum essential medium supplemented with 5% fetal bovine serum. Infectious PRRSV, LS-4, HM-1, HQ-5, GCH-3, GC-2, HQ-6 and ST-7 strains from Shijiazhuang of Hebei province (Additional file 10), were isolated in our laboratory at National Center of Wildlife Born Diseases, by inoculation of the sera or the tissue homogenates into SAM or MARC-145 cells.

### RNA extraction, reverse transcriptase PCR (RT-PCR) and nucleotide sequencing

RNAs were extracted from 200 μl of the culture supernatant of the PRRSV-infected SAM or MARC-145 cells using QIAamp^® ^viral RNA mini kit (Qiagen) according to the manufacturer's recommendation. Each target gene was amplified using QIAGEN^® ^One-Step RT-PCR kit (Qiagen). PCR and sequencing primers were shown as Table 1. The PCR reactions were done in a total volume of 25 μl containing 1 ng of the extracted cDNA,,200 μM of each (dNTP) (TakaRa), 1 × PCR buffer (TakaRa), 3.0 mM MgCl_2_, and 2.5 U of *Taq *polymerase(TakaRa). The PCR conditions were set as initial denaturation step at 94°C for 3 min followed by 40 cycles, each consisted of denaturation step at 94°C for 1 min, annealing step at 55°C for 1 min and elongation step at 72°C for 2 min, a final extensition at 72°C for 10 min was included. Size of amplicons was verified by agarose gel electrophoresis in TAE buffer using known standards. PCR products were purified using QIAquick^® ^PCR purification kit (Qiagen) and submitted to Invitrogen for sequencing.

**Table 1:** Primers used for PCR amplification of ORF2--ORF5 and NSP2 from PRRSV

Genes	Primer sequence
ORF2	5'-ACGAAGCTTATGAAATGGGGTCTATG-3'
	5'-TATCTCGAGTCACCGTGAGTTCAAAAG-3'
ORF3	5'-TTCATGATTTTCAGCAATGGCTAA-3'
	5'-GATGGTGATGTACACGGGCGT-3'
ORF4	5'-ACGGCGGCAATTGGTTTCACCTA-3'
	5'-CGTGGTCAAGCATTTCCCCAACATA-3'
ORF5	5'-CCTGAGACCATGAGGTGGGG-3'
	5'-TTTAGGGCATATATCATCACTGG-3'
NSP2	5'-TGAYTGGRATGTTGTGCTYCCTGG-3'
	5'-ATGCGAGARAGCCAYTCCTGCGT-3'

### Construction of phylogenetic trees

Nucleotide BLASTn analysis http://www.ncbi.nlm.nih.gov/BLAST was used to identify related genes of the viruses, and the reference sequences were obtained from GenBank. Pair-wise sequence alignments were also performed with the MEGA4.0 program [51]http://www.megasoftware.net/ to determine nucleotide sequence similarities. Alignments of each virus sequence were generated using program ClustalW [52]http://clustalw.genome.ad.jp/. Phylogenetic analyses of the aligned sequences for 5 gene segments (ORF2-5 and NSP2) were performed by the neighbor-joining method with 1000 bootstraps and Maximum-Likelihood with 100 bootstraps by using PHYLIP version 3.67 http://evolution.gs.washington.edu/phylip.html. All gene accession number of the isolates and other references virus were shown as Additional file 11.

### Comparison and analysis of amino acid sequences in gp2, gp3, gp4, gp5 and nsp2

Amino acid sequences of Chinese isolate virus (BJ-4), VR2332 and MLV gp2, gp3, gp4 and gp5 proteins were retrieved from the public domain database Entrez Protein, and compared each of them with all the 7 isolate virus proteins using the software ClustalW [52].

## Authors' contributions

HXH and CMW conceived the project. JL and HMZ conducted cell culture and isolation of PRRSV. BW and SA conducted data analysis and construction of phylogenetic trees. YHG and GYD conducted RNA extraction, reverse transcriptase PCR (RT-PCR) and nucleotide sequencing. WCM, BHZ and HHX wrote the paper. All authors read and approved the final manuscript. The authors declare no conflict of interest.

## Supplementary Material

Additional file 1 Table S1: Estimates of Evolutionary Divergence between isolates and references based on gp5 gene Sequences. Click here for file

Additional file 2 Table S2: Estimates of Evolutionary Divergence between isolates and references based on gp2 gene Sequences. Click here for file

Additional file 3 **Figure S1: Antigenic index analysis: plots of ORF2 generated by the Kyte and Doolittle method. Major areas of difference are indicated by arrows**. a, GC-2 was a representative of other two isolates because the same plots were shown for GC-2 and GCH-3. b, LS-4 was a representative of other two isolates because the same plots were shown for HM-1 and HQ-6. c, VR2332 was a representative of other three reference virus because the same plots were shown for BJ-4 and MLV. Click here for file

Additional file 4 Table S3: Estimates of Evolutionary Divergence between isolates and references based on gp3 gene Sequence. Click here for file

Additional file 5 **Figure S2. Antigenic index analysis plots of ORF3 generated by the Kyte and Doolittle method. Major areas of difference are indicated by arrows**. a, LS-4 was a representative of other six isolates because the same plots were shown for GC-2, ST-7, GCH-3, HM-1, HQ-5, HQ-6 and LS-4. b, VR2332 was a representative of other three reference virus because the same plots were shown for BJ-4 and MLV. Click here for file

Additional file 6 **Table S4. Estimates of Evolutionary Divergence between isolates and references based on gp4 gene Sequences.** Click here for file

Additional file 7 **Figure S3. antigenic index analysis: plots of ORF4 generated by the Kyte and Doolittle method. Major areas of difference are indicated by arrows**. a, LS-4 was a representative of other five isolates because of the same plots (GCH-3, HM-1, HQ-5, HQ-6 and ST-7). b, BJ-4 was a representative of other two reference virus because the same plots were shown for BJ-4 and MLV. Click here for file

Additional file 8 Table S5: Estimates of Evolutionary Divergence between isolates and references based on Nsp2 gene Sequences. Click here for file

Additional file 9 Table S6: prediction of immuno-dominant B-cell epitopes of NSP2 protein. Click here for file

Additional file 10 Table S7: The information of seven isolates from pig farms of Shijiazhuang city, in Hebei province. Click here for file

Additional file 11 Table S8: Summary of the PRRSV analyzed in this study. Click here for file
