# Supplementary material for: Phylogenetic analysis and molecular characteristics of seven variant Chinese field isolates of PRRSV
Source: BMC Microbiol. 2010 May 20;10:146. doi: 10.1186/1471-2180-10-146 (PMC2889949; doi:10.1186/1471-2180-10-146)
Supplement: Additional file 1 — Table S1: Estimates of Evolutionary Divergence between isolates and references based on gp5 gene Sequences. [file 1471-2180-10-146-S1.DOC]

**Additional file 1 Table S1. Estimates of Evolutionary Divergence between isolates and references based on gp5 gene Sequences**

|  | **LS-4** | **HM-1** | **HQ-5** | **GCH-3** | **GC-2** | **HQ-6** | **ST-7** | **VR2332** | **MLV** |
| --- | --- | --- | --- | --- | --- | --- | --- | --- | --- |
| **HM-1** | 0.005 |  |  |  |  |  |  |  |  |
| **HQ-5** | 0.005 | 0.01 |  |  |  |  |  |  |  |
| **GCH-3** | 0.005 | 0 | 0.01 |  |  |  |  |  |  |
| **GC-2** | 0.026 | 0.021 | 0.032 | 0.021 |  |  |  |  |  |
| **HQ-6** | 0.01 | 0.005 | 0.016 | 0.005 | 0.026 |  |  |  |  |
| **ST-7** | 0.016 | 0.01 | 0.021 | 0.01 | 0.032 | 0.016 |  |  |  |
| **VR2332** | 2.372 | 2.429 | 2.372 | 2.429 | 2.429 | 2.429 | 2.429 |  |  |
| **MLV** | 2.429 | 2.429 | 2.429 | 2.429 | 2.372 | 2.429 | 2.429 | 3.065 |  |
| **BJ-4** | 3.471 | 3.471 | 3.317 | 3.471 | 3.471 | 3.471 | 3.471 | 2.865 | 2.778 |
