# Supplementary figures and images for: Phylogenetic analysis and molecular characteristics of seven variant Chinese field isolates of PRRSV
Source: BMC Microbiol. 2010 May 20;10:146. doi: 10.1186/1471-2180-10-146 (PMC2889949; doi:10.1186/1471-2180-10-146)

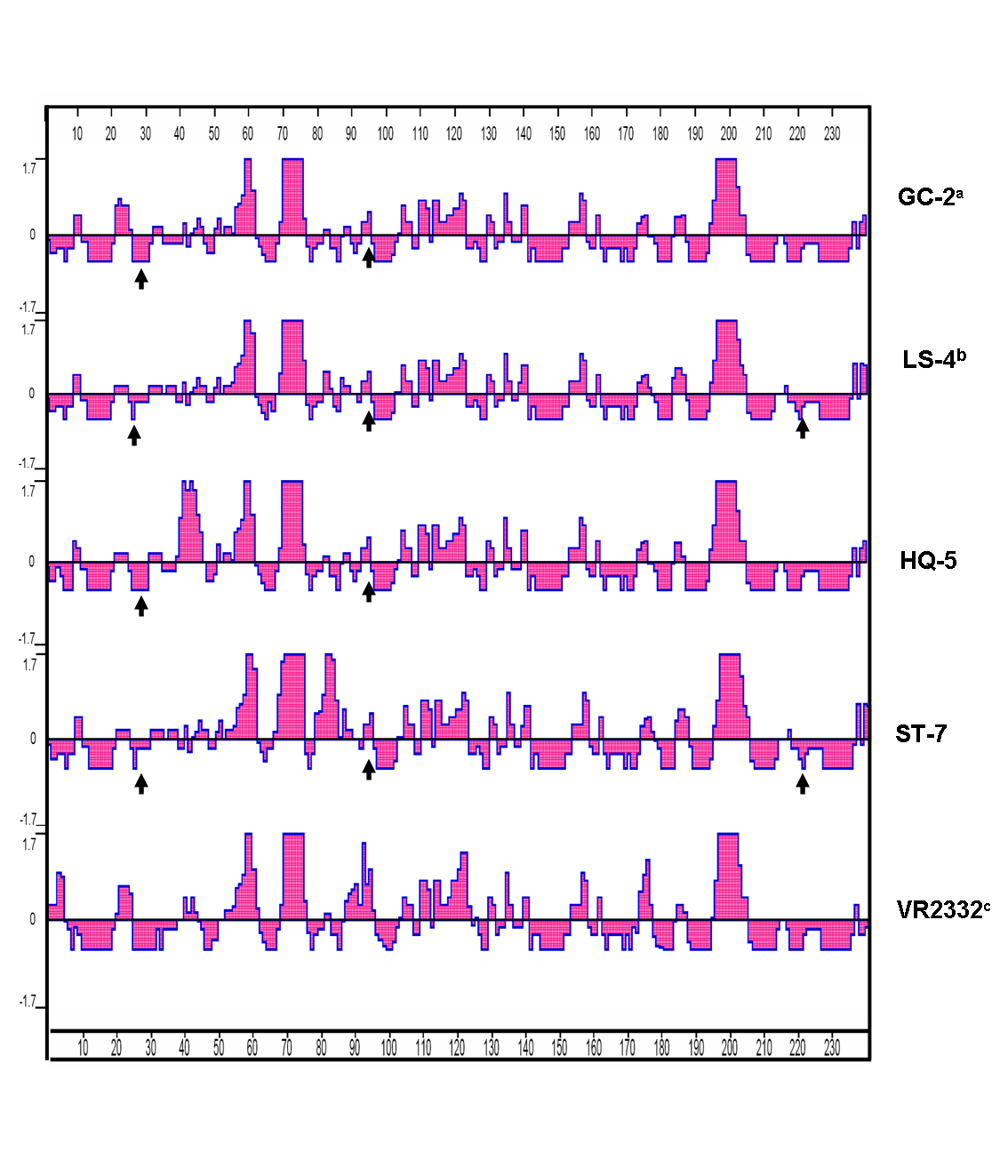

Supplement: Additional file 3 — Figure S1: Antigenic index analysis: plots of ORF2 generated by the Kyte and Doolittle method. Major areas of difference are indicated by arrows. a, GC-2 was a representative of other two isolates because the same plots were shown for GC-2 and GCH-3. b, LS-4 was a representative of other two isolates because the same plots were shown for HM-1 and HQ-6. c, VR2332 was a representative of other three reference virus because the same plots were shown for BJ-4 and MLV. [file 1471-2180-10-146-S3.TIFF]

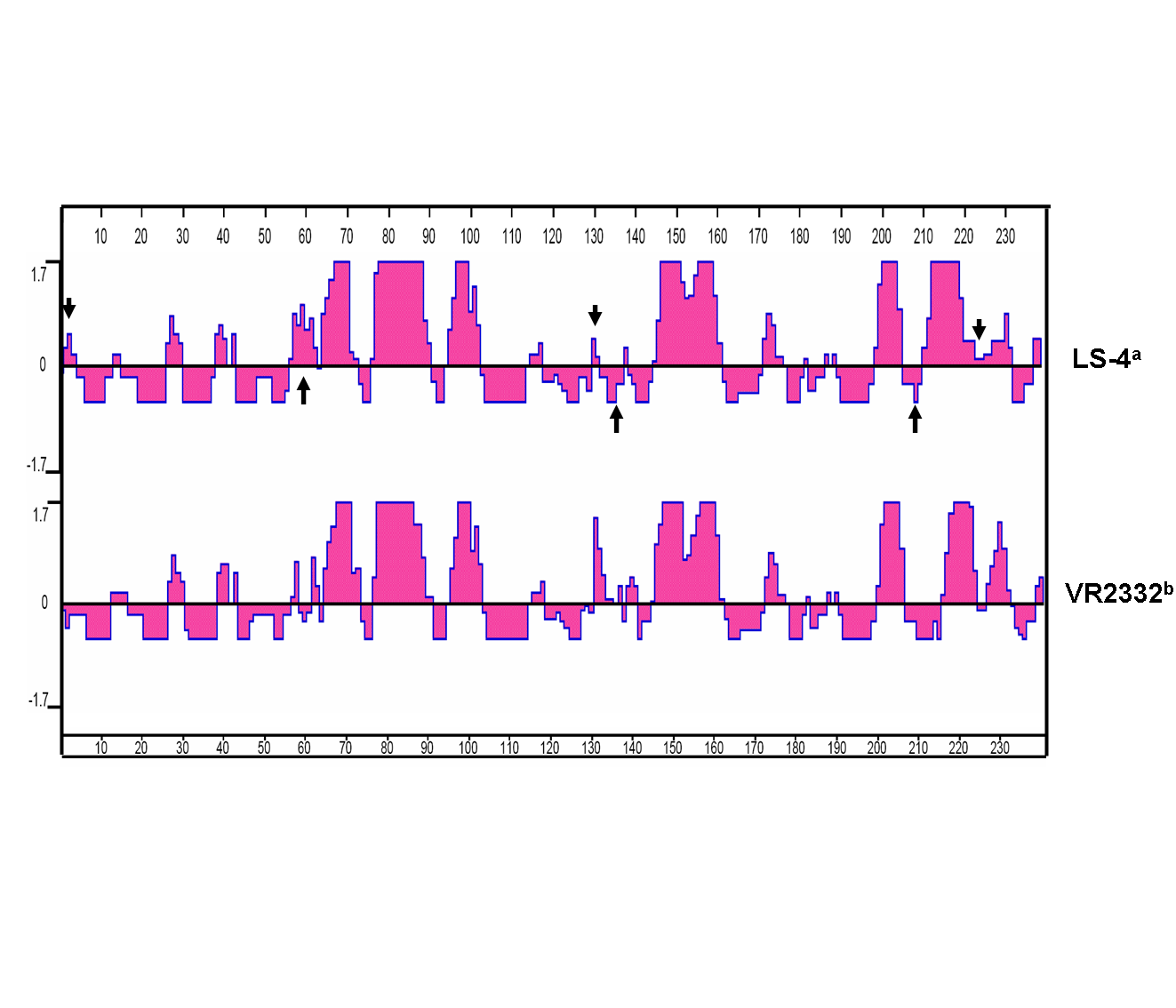

Supplement: Additional file 5 — Figure S2. Antigenic index analysis plots of ORF3 generated by the Kyte and Doolittle method. Major areas of difference are indicated by arrows. a, LS-4 was a representative of other six isolates because the same plots were shown for GC-2, ST-7, GCH-3, HM-1, HQ-5, HQ-6 and LS-4. b, VR2332 was a representative of other three reference virus because the same plots were shown for BJ-4 and MLV. [file 1471-2180-10-146-S5.TIFF]

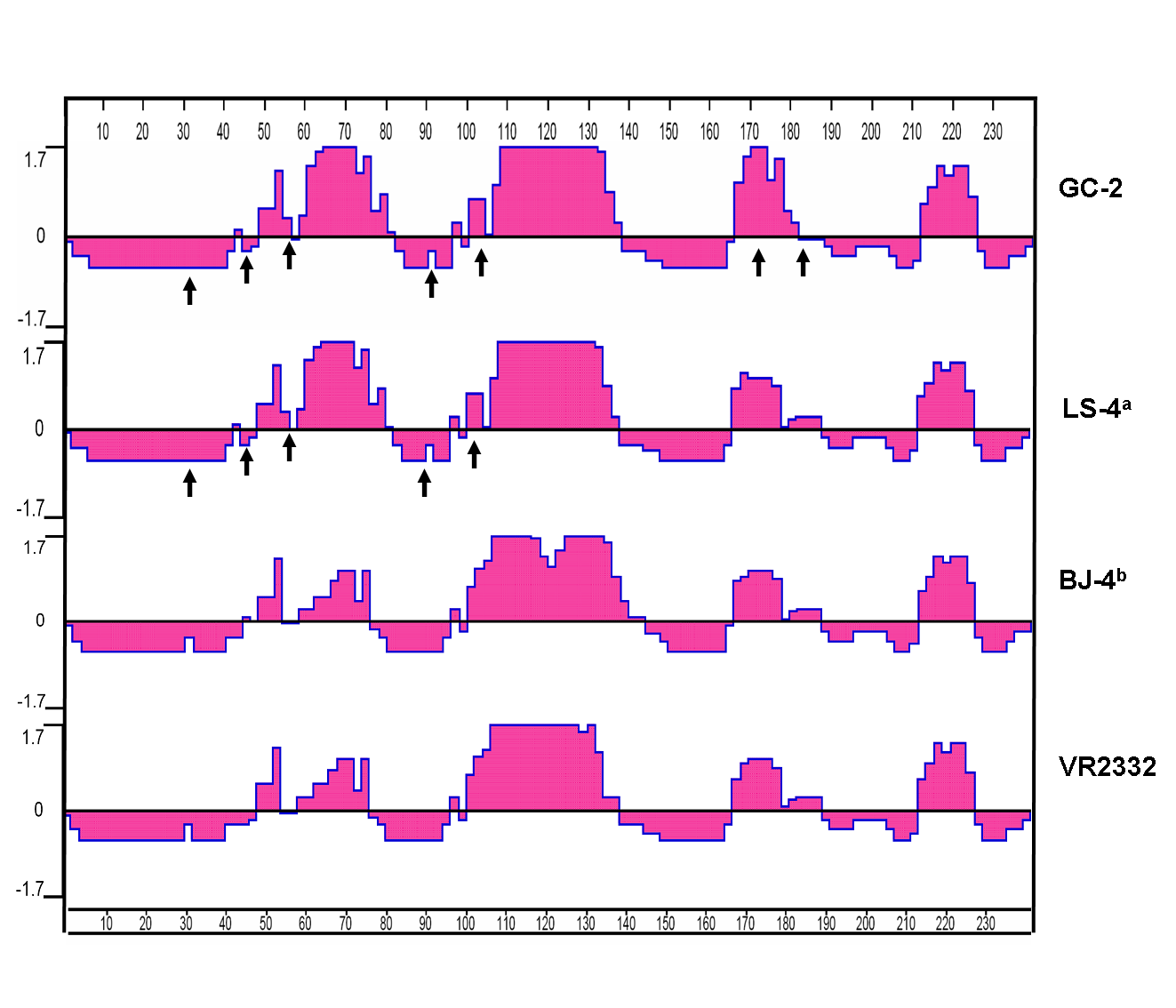

Supplement: Additional file 7 — Figure S3. antigenic index analysis: plots of ORF4 generated by the Kyte and Doolittle method. Major areas of difference are indicated by arrows. a, LS-4 was a representative of other five isolates because of the same plots (GCH-3, HM-1, HQ-5, HQ-6 and ST-7). b, BJ-4 was a representative of other two reference virus because the same plots were shown for BJ-4 and MLV. [file 1471-2180-10-146-S7.TIFF]
