# Supplementary material for: Phylogenetic analysis and molecular characteristics of seven variant Chinese field isolates of PRRSV
Source: BMC Microbiol. 2010 May 20;10:146. doi: 10.1186/1471-2180-10-146 (PMC2889949; doi:10.1186/1471-2180-10-146)
Supplement: Additional file 4 — Table S3: Estimates of Evolutionary Divergence between isolates and references based on gp3 gene Sequence. [file 1471-2180-10-146-S4.DOC]

**Additional file 4 Table S3. Estimates of Evolutionary Divergence between isolates and references based on gp3 gene Sequences**

|  | **LS-4** | **HM-1** | **HQ-5** | **GCH-3** | **GC-2** | **HQ-6** | **ST-7** | **BJ-4** | **VR2332** |
| --- | --- | --- | --- | --- | --- | --- | --- | --- | --- |
| **HM-1** | 0.02 |  |  |  |  |  |  |  |  |
| **HQ-5** | 0.02 | 0.016 |  |  |  |  |  |  |  |
| **GCH-3** | 0.032 | 0.028 | 0.02 |  |  |  |  |  |  |
| **GC-2** | 0.02 | 0.016 | 0.008 | 0.02 |  |  |  |  |  |
| **HQ-6** | 0.02 | 0.016 | 0.008 | 0.02 | 0 |  |  |  |  |
| **ST-7** | 0.008 | 0.012 | 0.012 | 0.024 | 0.012 | 0.012 |  |  |  |
| **BJ-4** | 0.144 | 0.144 | 0.144 | 0.157 | 0.144 | 0.144 | 0.144 |  |  |
| **VR2332** | 0.144 | 0.144 | 0.144 | 0.157 | 0.144 | 0.144 | 0.144 | 0.008 |  |
| **MLV** | 0.144 | 0.144 | 0.144 | 0.157 | 0.144 | 0.144 | 0.144 | 0 | 0.008 |
