# Supplementary material for: Phylogenetic analysis and molecular characteristics of seven variant Chinese field isolates of PRRSV
Source: BMC Microbiol. 2010 May 20;10:146. doi: 10.1186/1471-2180-10-146 (PMC2889949; doi:10.1186/1471-2180-10-146)
Supplement: Additional file 6 — Table S4. Estimates of Evolutionary Divergence between isolates and references based on gp4 gene Sequences. [file 1471-2180-10-146-S6.DOC]

**Additional file 6 Table S4. Estimates of Evolutionary Divergence between isolates and references based on gp4 gene Sequences**

|  | **LS-4** | **HM-1** | **HQ-5** | **GCH-3** | **GC-2** | **HQ-6** | **ST-7** | **BJ-4** | **VR2332** |
| --- | --- | --- | --- | --- | --- | --- | --- | --- | --- |
| **HM-1** | 0 |  |  |  |  |  |  |  |  |
| **HQ-5** | 0 | 0 |  |  |  |  |  |  |  |
| **GCH-3** | 0 | 0 | 0 |  |  |  |  |  |  |
| **GC-2** | 0.006 | 0.006 | 0.006 | 0.006 |  |  |  |  |  |
| **HQ-6** | 0 | 0 | 0 | 0 | 0.006 |  |  |  |  |
| **ST-7** | 0.011 | 0.011 | 0.011 | 0.011 | 0.017 | 0.011 |  |  |  |
| **BJ-4** | 0.102 | 0.102 | 0.102 | 0.102 | 0.108 | 0.102 | 0.114 |  |  |
| **VR2332** | 0.095 | 0.095 | 0.095 | 0.095 | 0.102 | 0.095 | 0.108 | 0.011 |  |
| **MLV** | 0.095 | 0.095 | 0.095 | 0.095 | 0.102 | 0.095 | 0.108 | 0.006 | 0.006 |
