# Supplementary material for: Phylogenetic analysis and molecular characteristics of seven variant Chinese field isolates of PRRSV
Source: BMC Microbiol. 2010 May 20;10:146. doi: 10.1186/1471-2180-10-146 (PMC2889949; doi:10.1186/1471-2180-10-146)
Supplement: Additional file 8 — Table S5: Estimates of Evolutionary Divergence between isolates and references based on Nsp2 gene Sequences. [file 1471-2180-10-146-S8.DOC]

**Additional file 8 Table S5. Estimates of Evolutionary Divergence between isolates and references based on Nsp2 gene Sequences**

|  | **LS-4** | **HM-1** | **HQ-5** | **GCH-3** | **GC-2** | **HQ-6** | **ST-7** | **BJ-4** | **VR2332** |
| --- | --- | --- | --- | --- | --- | --- | --- | --- | --- |
| **HM-1** | 0.021 |  |  |  |  |  |  |  |  |
| **HQ-5** | 0.021 | 0.017 |  |  |  |  |  |  |  |
| **GCH-3** | 0.023 | 0.017 | 0.002 |  |  |  |  |  |  |
| **GC-2** | 0.021 | 0.016 | 0.013 | 0.013 |  |  |  |  |  |
| **HQ-6** | 0.02 | 0.016 | 0.018 | 0.018 | 0.017 |  |  |  |  |
| **ST-7** | 0.027 | 0.023 | 0.026 | 0.026 | 0.024 | 0.017 |  |  |  |
| **BJ-4** | 0.275 | 0.281 | 0.28 | 0.283 | 0.278 | 0.276 | 0.28 |  |  |
| **VR2332** | 0.275 | 0.28 | 0.278 | 0.281 | 0.278 | 0.276 | 0.278 | 0.013 |  |
| **MLV** | 0.272 | 0.276 | 0.275 | 0.278 | 0.275 | 0.273 | 0.275 | 0.007 | 0.008 |
