# Supplementary material for: Phylogenetic analysis and molecular characteristics of seven variant Chinese field isolates of PRRSV
Source: BMC Microbiol. 2010 May 20;10:146. doi: 10.1186/1471-2180-10-146 (PMC2889949; doi:10.1186/1471-2180-10-146)
Supplement: Additional file 9 — Table S6: prediction of immuno-dominant B-cell epitopes of NSP2 protein. [file 1471-2180-10-146-S9.DOC]

**Additional file 9 Table S6. Prediction of immuno-dominant B-cell epitopes of NSP2 protein**

| virus strains | Predicted B cell epitopes  (AA Position) | | | | |
| --- | --- | --- | --- | --- | --- |
| LS-4 | 29-49 | 480-488 | 495-511 | 515-516 | 768-863 |
| HM-1 | 29-49 | 480-511 |  | 515-516 | 768-863 |
| HQ-5 | 29-49 | 480-511 |  | 515-516 | 773-863 |
| GCH-3 | 29-49 | 480-511 |  | 515-516 | 773-863 |
| GC-2 | 29-49 | 480-511 |  | 515-516 | 768-863 |
| HQ-6 | 29-49 | 480-511 |  | 515-516 | 768-863 |
| ST-7 | 29-49 | 480-510 |  |  | 773-863 |
