# Supplementary material for: Phylogenetic analysis and molecular characteristics of seven variant Chinese field isolates of PRRSV
Source: BMC Microbiol. 2010 May 20;10:146. doi: 10.1186/1471-2180-10-146 (PMC2889949; doi:10.1186/1471-2180-10-146)
Supplement: Additional file 10 — Table S7: The information of seven isolates from pig farms of Shijiazhuang city, in Hebei province. [file 1471-2180-10-146-S10.DOC]

**Additional file 10 Table S7. The information of seven isolates from pig farms of Shijiazhuang city, in Hebei province**

| **Name of isolates** | [**Pathogenicity**](dict://key.0895DFE8DB67F9409DB285590D870EDD/pathogenicity)**^1^** | | | [**Pig farm**](dict://key.0895DFE8DB67F9409DB285590D870EDD/pig farm) | **Region** | **Province** |
| --- | --- | --- | --- | --- | --- | --- |
|  | TCID_50_ (Marc-145) | Mortality of Piglet(N=5) | Clinical signs^2^ |  |  |  |
| LS-4 | 10^-4.5^ | 100% | Obvious clinical signs | LS farm | Shijiazhuang city | Hebei province |
| HM-1 | 10^-4.15^ | 80% | Obvious clinical signs | HM farm | Shijiazhuang city | Hebei province |
| HQ-5 | 10^-4.5^ | 100% | Obvious clinical signs | HQ farm | Shijiazhuang city | Hebei province |
| HQ-6 | 10^-4.5^ | 100% | Obvious clinical signs | HQ farm | Shijiazhuang city | Hebei province |
| GCH-3 | 10^-3.75^ | 60% | Obvious clinical signs | GCH farm | Shijiazhuang city | Hebei province |
| GC-2 | 10^-4.0^ | 80% | Obvious clinical signs | GC farm | Shijiazhuang city | Hebei province |
| ST-7 | 10^-4.0^ | 80% | Obvious clinical signs | ST farm | Shijiazhuang city | Hebei province |

Note: 1, TCID_50_ was tested by inoculating Marc-145 cells using Reed-Muench method; 40-day-old PRRSV-free piglets (5 per infection group) were inoculated intramuscularly with 10^5.0^ mean tissue cultures infectious doses/2mL, and then piglets in each group were observed for 21 days to evaluate death rates. All procedures were conducted in a BSL-3 facility and approved by the Chinese CADC ethics committee.

2, Obvious clinical signs, including inappetence, lethargy, high and continuous fever, red discolorations in the body.
