# Supplementary material for: Phylogenetic analysis and molecular characteristics of seven variant Chinese field isolates of PRRSV
Source: BMC Microbiol. 2010 May 20;10:146. doi: 10.1186/1471-2180-10-146 (PMC2889949; doi:10.1186/1471-2180-10-146)
Supplement: Additional file 11 — Table S8: Summary of the PRRSV analyzed in this study. [file 1471-2180-10-146-S11.DOC]

| **Name of The isolate** | **GP4** | **GP3** | **GP2** | **NSP2** | **GP5** |
| --- | --- | --- | --- | --- | --- |
| LS-4 | EU017512.1 | EU017511.1 | EU017510.1 | EU075304.2 | EU075303 |
| HM-1 | EU177105.1 | EU177104.1 | EU177103.1 | EU177102.1 | EU177106 |
| HQ-5 | EU177110.1 | EU177109.1 | EU177108.1 | EU255920.1 | EU439252 |
| GCH-3 | EU177119.1 | EU177118.1 | EU177117.1 | EU669820.1 | EU177120 |
| GC-2 | EU177113.1 | EU255923.1 | EU255922.1 | EU255919.1 | EU177114 |
| HQ-6 | EU255926.1 | EU366149.1 | EU642603.1 | EU653014.1 | EU255925 |
| ST-7 | EU366150.1 | EU439254.1 | EU653015.1 | EU642604.1 | EU366151 |
| HB-3(cz) | - | - | - | - | EU478435 |
| BJ-4 | AF331831 | AF331831 | AF331831 | AF331831 | AF331831 |
| HUB2 | EF112446 | EF112446 | EF112446 | EF112446 | EF112446 |
| CC-1 | EF153486 | EF153486 | EF153486 | EF153486 | EF153486 |
| HuN | EF517962 | EF517962 | EF517962 | EF517962 | EF517962 |
| HuN829 | - | - | - | - | [EU399853](http://www.ncbi.nlm.nih.gov/entrez/viewer.fcgi?db=nuccore&id=166079258) |
| TJZHJ3 | - | - | - | - | EU213143 |
| BJsy06 | EU097707 | EU097707 | EU097707 | EU097707 | EU097707 |
| HKEU16 | EU076704 | EU076704 | EU076704 | EU076704 | EU076704 |
| SHH | EU106888 | EU106888 | EU106888 | EU106888 | EU106888 |
| LN | EU109502 | EU109502 | EU109502 | EU109502 | EU109502 |
| WUH1 | EU187484 | EU187484 | EU187484 | EU187484 | EU187484 |
| WUH2 | - | - | - | - | EU678352 |
| Jiangxi-3 | EU200961.1 | EU200961.1 | EU200961.1 | EU200961.1 | EU200961 |
| Henan-1 | EU200962.1 | EU200962.1 | EU200962.1 | EU200962.1 | EU200962 |
| HeNYE4 | - | - | - | - | EU399837 |
| XH-GD | EU624117 | EU624117 | EU624117 | EU624117 | EU624117 |
| GZZB | - | - | - | EU140617.1 | EU140611.1 |
| GZJL | - | - | - | - | FJ947000.1 |
| GUIZHOU-1 | - | - | - | - | EU259060.1 |
| HLJZY | - | - | - | - | [EU213119](http://www.ncbi.nlm.nih.gov/entrez/viewer.fcgi?db=nuccore&id=159135326) |
| YNYX3 | - | - | - |  | EU213145 |
| YN08 | - | - | - | FJ361898.1 | FJ361889.1 |
| Hainan-2 | - | - | - | EU213088.1 | EF398052 |
| Hainan-1 | - | - | - | EU213089.1 | EF398051 |
| FJ-1 | [AY881994](http://www.ncbi.nlm.nih.gov/entrez/viewer.fcgi?db=nucleotide&val=60280505) | [AY881994](http://www.ncbi.nlm.nih.gov/entrez/viewer.fcgi?db=nucleotide&val=60280505) | [AY881994](http://www.ncbi.nlm.nih.gov/entrez/viewer.fcgi?db=nucleotide&val=60280505) | - | [AY881994](http://www.ncbi.nlm.nih.gov/entrez/viewer.fcgi?db=nucleotide&val=60280505) |
| GD | EU825724.1 | EU825724.1 | EU825724.1 | EU825724.1 | EU825724.1 |
| CH-1a | AY032626 | AY032626 | AY032626 | AY032626 | AY032626 |
| CH-1R | EU807840.1 | EU807840.1 | EU807840.1 | EU807840.1 | EU807840.1 |
| HZ061226 |  |  |  | EU595681.1 | EU595690.1 |
|  |  |  |  |  |  |
| JSyx | EU939312 | EU939312 | EU939312 | EU939312 | EU939312 |
| ZJJ07 | - | - | - | - | [EU709846](http://www.ncbi.nlm.nih.gov/entrez/viewer.fcgi?db=nuccore&id=188531989) |
| ZJJ05 | - | - | - | - | [EU709845](http://www.ncbi.nlm.nih.gov/entrez/viewer.fcgi?db=nuccore&id=188531987) |
| ZJ06 | - | - | - | - | EU709843 |
| GXHP-6 | - | - | - | - | EU562181 |
| GXHP-5 | - | - | - | - | EU428819 |
| SX071226 | - | - | - | EU595686.1 | EU595695 |
| AHES3 | - | - | - | - | EU399870 |
| AHZHP | - | - | - | - | EU399865 |
| SHX | - | - | - | - | EU480721 |
| SC07 | - | - | - | - | EU709834 |
| XJ07 | - | - | - | - | EU709841 |
| S1 | AF090173 | AF090173 | AF090173 | AF090173 | AF090173 |
| ZX07 | - | - | - | - | EU709847 |
| SD-1 | - | - | - | - | AY747596 |
| F1 | AF030306.1 | AF030306.1 | AF030306.1 | EU075304.2 | AF030306 |
| B13 | - | - | - | - | AY633973 |
| LV | M96262 | M96262 | M96262 | M96262 | M96262 |
| LMY | DQ473474 | DQ473474 | DQ473474 | DQ473474 | DQ473474 |
| VR2332 | EF536003 | EF536003 | EF536003 | EF536003 | EF536003 |
| MLV Resp | AF159149 | AF159149 | AF159149 | AF159149 | AF159149 |
| 02CB13 | - | - | - | - | AY297114 |
| NVSL 97-7895 | - | - | - | - | AY545985 |
